# Supercapacitor Performance of Magnetite Nanoparticles Enhanced by a Catecholate Dispersant: Experiment and Theory

**DOI:** 10.3390/molecules28041562

**Published:** 2023-02-06

**Authors:** Coulton Boucher, Oleg Rubel, Igor Zhitomirsky

**Affiliations:** Department of Materials Science and Engineering, McMaster University, Hamilton, ON L8S4L7, Canada

**Keywords:** iron oxide, supercapacitor, catechol, density functional theory, adsorption, capacitance

## Abstract

The full potential of Fe_3_O_4_ for supercapacitor applications can be achieved by addressing challenges in colloidal fabrication of high active mass electrodes. Exceptional adsorption properties of catecholate-type 3,4-dihydroxybenzoic acid (DHBA) molecules are explored for surface modification of Fe_3_O_4_ nanoparticles to enhance their colloidal dispersion as verified by sedimentation test results and Fourier-transform infrared spectroscopy measurements. Electrodes prepared in the presence of DHBA show nearly double capacitance at slow charging rates as compared to the control samples without the dispersant or with benzoic acid as a non-catecholate dispersant. Such electrodes with active mass of 40 mg cm^−2^ show a capacitance of 4.59 F cm^−2^ from cyclic voltammetry data at a scan rate of 2 mV s^−1^ and 4.72 F cm^−2^ from galvanostatic charge–discharge data at a current density of 3 mA cm^−2^. Experimental results are corroborated by density functional theory (DFT) analysis of adsorption behaviour of DHBA and benzoic acid at the (001) surface of Fe_3_O_4_. The strongest adsorption energy (ca. −1.8 eV per molecule) is due to the catechol group of DHBA. DFT analysis provides understanding of the basic mechanism of DHBA adsorption on the surface of nanoparticles and opens the way for fabrication of electrodes with high capacitance.

## 1. Introduction

Surface modification of materials using molecules from the catechol family is emerging as a new area of technological and scientific interest [1,2]. Catecholate molecules can achieve extremely strong adhesion to inorganic surfaces by utilizing a similar adsorption mechanism to that of mussel proteins when bonded to different surfaces [2,3,4]. Very fast and very strong adsorption to various inorganic substrates is a necessity for mussels to avoid damage by sea waves. This adhesion mechanism is attributed to bonding of the catechol ligands of the mussel proteins to metal atoms on material surfaces [4,5,6,7,8].

Polymer molecules were modified with catechol ligands for the fabrication of adherent polymer and composite films [4,9,10,11,12]. Catecholate molecules were used as charged dispersants for electrophoretic deposition of inorganic and organic materials [5,13,14]. Catecholate molecules were also utilized as particle transfer vehicles for liquid–liquid extraction [15], which prevented nanoparticle agglomeration. In this technology, inorganic particles were modified with catechol ligands in the synthesis medium or at the liquid–liquid interface and transferred from the synthesis medium directly to the device processing medium [15]. Catechol molecules facilitated efficient and versatile extraction [16] of MnO_2_, Mn_3_O_4_, FeOOH, and ZnO for energy storage and sensor applications. The phase transfer of the particles resulted in reduced agglomeration, which allowed for improved electrolyte access to the particle surface [16] and improved device performance.

Recent studies highlighted benefits of catecholate molecules for surface modification of materials for applications in dye-sensitized solar cells and photoelectrochemical sensors [17,18,19]. For dye-sensitized solar cells, a staggered gap heterojunction can occur at a catechol-TiO_2_ interface [17], which facilitates electron injection into the TiO_2_ semiconductor [20]. Such configurations improved dye-sensitized solar cell efficiency. Redox properties of catechol, related to oxidation of their phenolic OH groups, were used for electron transfer mediation in electrochemical biosensors [21]. Moreover, redox properties of catechol are under investigation for applications in bioelectronics [19,22], catalysis [23], and energy storage devices [24,25]. Enhanced capacitance has been seen in catechol-modified carbon cloth electrodes due to the additional contribution of electrochemical oxidation of catechol OH groups [26].

The ability of catechol to oxidize electrochemically to o-benzoquinone allows for many uses in electropolymerization [27,28]. It is known that o-benzoquinone participates in polymerization reactions, including chemical oxidative polymerization [29], electrochemical oxidative polymerization [30], and bipolar electropolymerization [27], to be used in a variety of biomedical applications including targeted drug delivery to cancer cells [31], catechol-modified polymers with antimicrobial properties [32], and structural adhesives for tissue and bone [33]. Catechol molecules can play an additional role as charge-transfer mediators [34]. The use of catecholate dopants for polypyrrole facilitated charge transfer, reduced electropolymerization potential, and allowed the fabrication of adherent polypyrrole films on non-noble substrates [35,36,37].

The functionalization of ferrimagnetic nanoparticles with catecholate molecules induced improved magnetization [38]. Other investigations reported applications of catecholate molecules for the fabrication of quantum dots and luminescent materials [39,40]. Catecholates have generated significant interest for applications in nanotechnology as capping, reducing, and structure directing additives, which facilitated the fabrication of nanoparticles of metals and metal oxides with controlled size, shape, and functional properties [41,42,43,44,45]. Further progress in the application of catecholates in nanotechnology can result in the development of advanced functional nanomaterials for modern applications. Catecholates are of particular interest for the fabrication of devices, based on materials, combining advanced magnetic and electrical properties [41,46,47].

This investigation was motivated by interesting electrical charge storage properties of iron oxides, such as Fe_2_O_3_ and Fe_3_O_4_ [41,48]. Fe_3_O_4_ is of particular interest for the development of multifunctional devices, based on the high capacitance and advanced ferrimagnetic properties of this material. It is known that Fe_3_O_4_ exhibits higher conductivity compared to many other spinel ferrimagnetic materials [49]. The relatively high conductivity of Fe_3_O_4_ is beneficial for electrochemical charge–discharge processes [49]. The pseudocapacitive charging mechanism of this material, containing Fe^2+^ and Fe^3+^ ions (Fe^2+^Fe_2_^3+^O_4_), is attributed to redox Fe^3+^ ↔ Fe^2+^ reactions. The charge storage properties in the positive potential range [50,51] are attributed to oxidation of Fe^2+^ ions (Fe^2+^ → Fe^3+^), whereas the charging mechanism in the negative potential range is based on reduction of Fe^3+^ ions (Fe^3+^ → Fe^2+^) [41,52,53]. Fe_3_O_4_ is especially important for the development of negative electrodes of advanced asymmetric supercapacitors with an enlarged voltage window. Recently, significant progress was achieved in the development of different materials for positive electrodes, which showed high capacitance [54,55,56]. However, lower capacitance of the negative electrodes, such as activated carbon, is a limiting factor in the development of advanced devices. The important tasks in the development of Fe_3_O_4_ electrodes are the fabrication of small nanoparticles and prevention of their agglomeration, which can result from Van der Waals forces and magnetic attraction forces. Moreover, efficient mixing of non-agglomerated and well-dispersed nanoparticles with conductive additives is necessary for good electrode performance. Therefore, advanced capping and dispersing agents are critically important for the synthesis of small non-agglomerated Fe_3_O_4_ nanoparticles and fabrication of composite electrodes.

**Figure 1:**
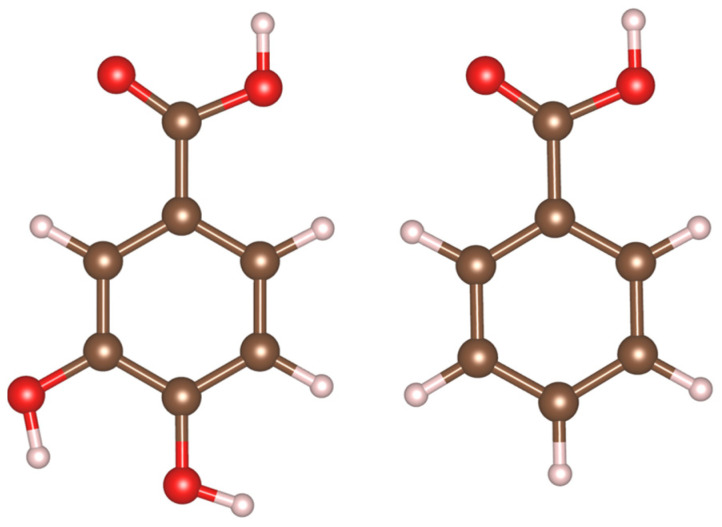
Chemical structure of 3,4-dihydroxybenzoic acid (**left**) and benzoic acid (**right**).

The goal of this investigation is the fabrication of Fe_3_O_4_ electrodes for supercapacitors using a catecholate-type 3,4-dihydroxybenzoic acid (DHBA) as a capping and dispersing agent for Fe_3_O_4_ nanoparticles. This goal was achieved in experimental studies of the synthesis of Fe_3_O_4_ particles, fabrication, and testing of supercapacitor electrodes and application of density functional theory (DFT) [57] for the analysis of the DHBA adsorption mechanism. Fe_3_O_4_ has emerged as a promising supercapacitor material due to its low cost and environmentally benign nature [58]. Here, we aimed at improving electrochemical performance of fabricated Fe_3_O_4_ supercapacitor anodes and selected DHBA as a catechol molecule to play the role of a capping and dispersing agent. The chemical structure of DHBA contains adjacent phenolic OH functional groups on the 3rd and 4th carbons on the benzene ring (Figure 1), providing strong adsorption to the particle surface, while a carboxyl group can facilitate dispersion via electrostatic repulsion [59,60]. These adsorption and dispersion properties are critical to prevent agglomeration of nanoparticles in solution, particularly materials with magnetic properties, such as Fe_3_O_4_. Benzoic acid (BA) is chosen as a negative control in our experimental design. The chemical structures of DHBA and BA differ only by the adjacent phenolic OH groups present in DHBA but not in BA (Figure 1). To corroborate the understanding of the adsorption processes seen in our experimental results, we used DFT to model DHBA and BA adsorption on the surface of Fe_3_O_4_ and assess their binding affinities and mechanisms.

## 2. Results and Discussion

### 2.1. Experimental Results

**Figure 2:**
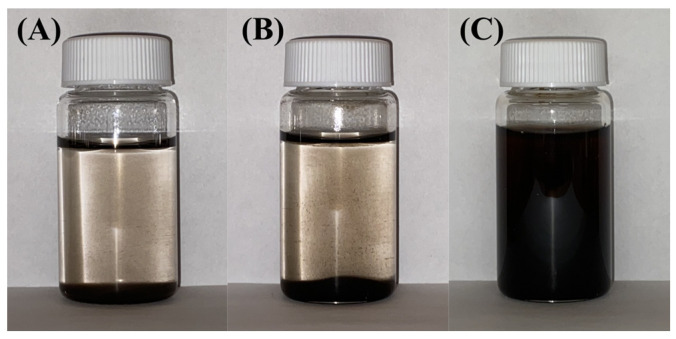
Fe_3_O_4_ suspension in water with (**A**) no dispersing agent, (**B**) BA as a dispersing agent, and (**C**) DHBA as a dispersing agent 1 h after ultrasonication.

Dispersing properties of BA and DHBA were investigated by analysing the suspension stability of Fe_3_O_4_ particles in water (Figure 2). Fe_3_O_4_ suspensions without additives and containing BA showed rapid precipitation. However, the suspension prepared with DHBA as a dispersing agent remained stable, further confirming the role of the phenolic OH groups of the molecule for adsorption, which are functional groups that BA lacks. The synthesis of Fe_3_O_4_ was conducted at pH = 9, which is above the isoelectric point of Fe_3_O_4_ (pH = 6.5) [61]. Therefore, the as-prepared Fe_3_O_4_ particles were negatively charged. The adsorption of anionic DHBA on the negatively charged Fe_3_O_4_ particles indicated a strong adsorption power of the catecholate DHBA molecules.

**Figure 3:**
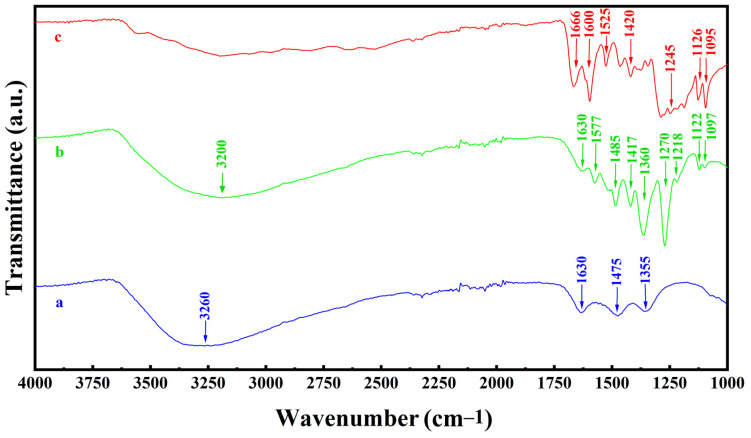
FTIR spectra of (a) Fe_3_O_4_ prepared without DHBA, (b) Fe_3_O_4_ prepared using DHBA, and (c) pure as-received DHBA.

The adsorption of DHBA on Fe_3_O_4_ particles was confirmed by results of Fourier Transform Infrared (FTIR) spectroscopy studies. Figure 3 compares FTIR spectra of Fe_3_O_4_ prepared without and with DHBA and pure DHBA.

The FTIR spectrum of Fe_3_O_4_ prepared without DHBA (Figure 3a) showed absorptions at 1355, 1475, and 1630 cm^−1^, which can be attributed [13,62] to stretching of surface OH groups and vibrations of adsorbed CO_2_. The broad absorption centred at 3260 cm^−1^ resulted from the O-H bending vibrations of adsorbed water [62]. Similar absorptions were observed in the spectrum of Fe_3_O_4_ prepared using DHBA (Figure 3b). However, the spectrum of Fe_3_O_4_ prepared using DHBA showed additional absorptions due to adsorbed DHBA. The absorptions at 1097 and 1122 cm^−1^ resulted from bending C-H vibrations [63,64]. Similar absorptions were observed in the spectrum of DHBA (Figure 3c). The C-O stretching and bending vibrations [7,64] of DHBA resulted in a broad absorption (Figure 3c) centred around 1245 cm^−1^ and corresponding absorptions at 1218 and 1270 cm^−1^ for Fe_3_O_4_ prepared using DHBA (Figure 3b), which were not observed in the spectrum of Fe_3_O_4_ prepared without DHBA (Figure 3a). FTIR studies also revealed additional peaks related to the C-C vibrations in the range of 1300–1600 cm^−1^ in the spectrum of Fe_3_O_4_ prepared using DHBA (Figure 3b), which were not observed in the spectrum of Fe_3_O_4_ prepared without DHBA (Figure 3a). Such peaks, related to C-C stretching of aromatic rings, were observed in the spectrum of pure DHBA (Figure 3c). Electrokinetic measurements showed that the adsorption of anionic DHBA resulted in increasing absolute value of negative zeta potential from −28.9 mV for Fe_3_O_4_ prepared without DHBA to −38.7 mV for Fe_3_O_4_ prepared using DHBA. Therefore, the results of sedimentation tests, zeta potential measurements, and FTIR spectroscopy indicated that DHBA adsorbed on Fe_3_O_4_ and facilitated the formation of suspensions with improved colloidal stability.

**Figure 4:**
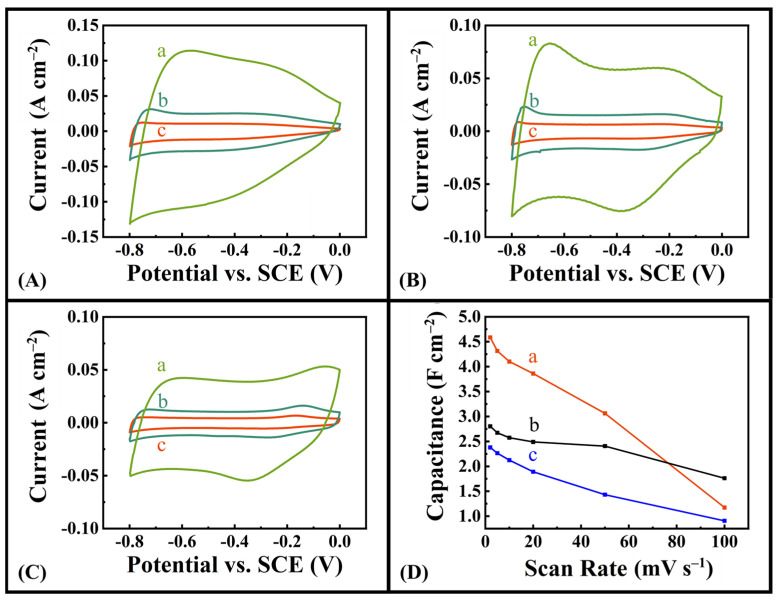
(**A**–**C**) CVs at scan rates of (a) 20, (b) 5, and (c) 2 mV s^−1^ for Fe_3_O_4_–MWCNT electrodes prepared with (**A**) DHBA as dispersing agent, (**B**) BA as dispersing agent, and (**C**) no dispersing agent. (**D**) Capacitance vs. scan rate for Fe_3_O_4_–MWCNT electrodes prepared with (a) DHBA as dispersing agent, (b) BA as dispersing agent, and (c) no dispersing agent.

In this investigation, MWCNT were used as a conductive additive for the fabrication of composite Fe_3_O_4_-MWCNT electrodes with enhanced electronic conductivity. Figure 4 presents experimental results for the CV analysis of the Fe_3_O_4_-MWCNT composite electrodes. Electrodes were fabricated with synthesized Fe_3_O_4_ and MWCNT in the presence of DHBA, BA, and no dispersing agent to investigate the impact of a dispersant on capacitive behaviour. Electrodes prepared using DHBA showed significant improvement in capacitance at scan rates 2–50 mV s^−1^. The role of the phenolic OH groups of DHBA is evident when comparing CVs and obtained capacitances of electrodes fabricated with DHBA and BA as dispersants. The capacitance of 4.59 F cm^−2^ for DHBA at 2 mV s^−1^ scan rate represents significant improvement, compared to the 2.38 F cm^−2^ and 2.80 F cm^−2^ obtained when using no dispersing agent and BA dispersant, respectively. The slightly lower capacitance of electrodes prepared using DHBA at a high scan rate of 100 mV s^−1^ can be attributed to the insulating properties of adsorbed DHBA, which affect the capacitance at such high scan rate.

**Figure 5:**
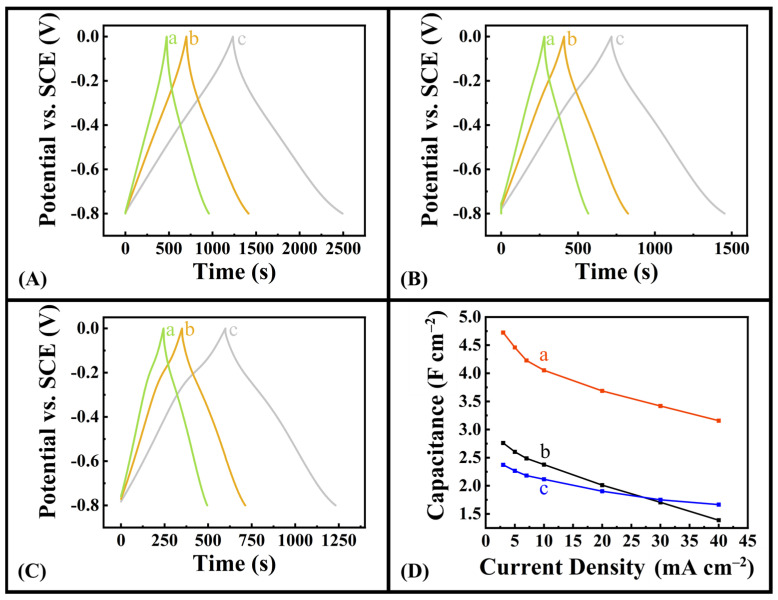
(**A**–**C**) charge–discharge curves at current densities of (a) 7 mA cm^−2^, (b) 5 mA cm^−2^, and (c) 3 mA cm^−2^ for Fe_3_O_4_–MWCNT electrodes prepared with (**A**) DHBA as dispersing agent, (**B**) BA as dispersing agent, and (**C**) no dispersing agent. (**D**) Capacitance vs. current density profiles for Fe_3_O_4_–MWCNT electrodes prepared with (a) DHBA as dispersing agent, (b) BA as dispersing agent, and (c) no dispersing agent.

Figure 5 compares the galvanostatic charge–discharge curves (A–C) for different electrodes, with corresponding capacitances (D) calculated from the discharge data at different current densities. GCD was performed in a voltage window of −0.8–0 V vs. SCE. Nearly symmetrical and triangular charge–discharge curves are seen, confirming the pseudocapacitive behaviour. Electrodes prepared with DHBA showed significant increase in discharge time and significant improvement in capacitance when compared to electrodes prepared using BA or no dispersant. A capacitance of 4.72 F cm^−2^ was obtained for electrodes made with DHBA at a current density of 3 mA cm^−2^ and decreased to 3.16 F cm^−2^ with increasing current density. This capacitance shows significant improvement compared to the capacitances of 2.76 F cm^−2^ and 2.37 F cm^−2^ obtained for the electrodes prepared with BA as a dispersing agent and no dispersing agent, respectively, at the current density of 3 mA cm^−2^. It should be noted that capacitance decreases with increasing charge–discharge time. The difference in the capacitance values obtained using CV and GCD data can result from the different time scale ranges used in both experiments. The Coulombic efficiency at 3 mA cm^−2^ was 98.0%; it decreased with current density to the value of 97.7% at a current density of 40 mA cm^−2^. The electrodes prepared using DHBA showed significantly higher capacitance, compared to electrodes prepared with BA and without dispersant in the current density range of 3–40 mA cm^−2^. The high areal capacitance obtained in this investigation for negative electrodes is important for the development of asymmetric devices operating in Na_2_SO_4_ electrolyte [55,56]. The obtained capacitance is higher than the capacitance of carbon electrodes of the same mass operating in the negative potential range [54]. It is comparable with capacitance of advanced positive electrodes [55,56]. It should be noted that catechol exhibits redox active properties in a positive potential range [25,65]. Specifically, the redox activity is associated with the transition of phenolic groups of DHBA to a quinone-like structure [66,67]. In this investigation, a catecholate type DHBA dispersant was used for the fabrication of negative electrodes. Therefore, redox reaction contribution of this dispersant to the to the electrode capacitance in the negative potential range is not expected. However, adsorbed catecholate molecules can facilitate charge transfer [21,35,36].

**Figure 6:**
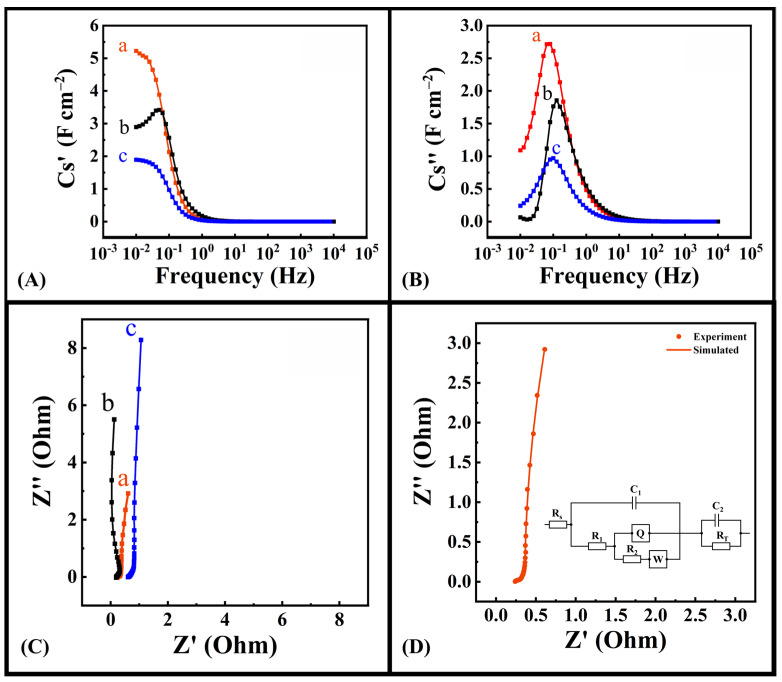
(**A**) Real part of capacitance (Cs′) vs. frequency, (**B**) imaginary part of capacitance (Cs″) vs. frequency, and (**C**) Nyquist plot for Fe_3_O_4_–MWCNT electrodes prepared with (a) DHBA as dispersing agent, (b) BA as dispersing agent, and (c) no dispersing agent, and (**D**) modelling of impedance for Fe_3_O_4_ electrode prepared using DHBA and corresponding equivalent circuit (inset).

The analysis of impedance data provides evidence of improved performance of the electrode fabricated using DHBA as a dispersing agent. Figure 6A shows significant increase in the low frequency capacitance (Cs′) for the electrode fabricated with DHBA as a dispersing agent compared to the electrodes where BA and no dispersing agent was used. The analysis of frequency dependences of C_S_″ shows an increase in the relaxation frequency for both electrodes prepared using DHBA and BA compared to the electrode prepared with no dispersing agent (Figure 6B). The Nyquist plot, presented in Figure 6C, shows relatively low real part of impedance (Z′), indicating low resistance. Furthermore, the large slope shown in the Z″ vs. Z′ curves indicate good capacitive behaviour. Modelling of impedance spectroscopy data using equivalent circuits is an important strategy for the analysis of supercapacitor electrodes [68]. In this investigation, an equivalent circuit developed for high active mass bulk electrodes [69] was used. The simulation results presented in Figure 6D showed good agreement with experimental data and indicated that the electrodes exhibited low charge transfer resistance (R_T_), which was found to be 0.08 Ohm.

Fe_3_O_4_ is a promising negative electrode material for the development of asymmetric supercapacitors [52] with enlarged voltage window for operation in mild electrolytes, such as K_2_SO_4_ or Na_2_SO_4_. However, the lower specific capacitance of the negative electrodes [52], compared to the capacitance of positive electrodes, limits the development of such devices. Another difficulty is related to significant decrease in specific capacitance with increasing active mass [70]. However, high active mass loading is necessary for practical applications.

The approach developed in this investigation allowed for improved utilization of capacitive properties of Fe_3_O_4_. Despite the high active mass of 40 mg cm^−2^, the capacitance of 114.8 F g^−1^ (4.59 F cm^−2^) was obtained from the CV data, which is higher than the reported capacitance [52] of 75 F g^−1^ at active mass of 8.8 mg cm^−2^.

### 2.2. DFT Modeling of Adsorption on Fe_3_O_4_ (001) Surface

Next, we performed DFT modelling of adsorption of molecules studied experimentally at the surface of Fe_3_O_4_. For this purpose, we established the most stable surface of Fe_3_O_4_ and its reconstruction. Relaxation of a bulk cubic Fe_3_O_4_ unit cell was conducted. The corresponding lattice parameter, bond lengths, and magnetic moments were in good agreement with values obtained in a prior DFT+U Fe_3_O_4_ study [71] and the experimental values found in literature. Table 1 summarizes these results, where differences between the two DFT studies may be attributed to the selection of a slightly different value for U_eff_, as well as the Van der Waals interaction, accounted for in our work but not in the prior study [71].

For the purposes of this study, adsorption investigations were performed exclusively on the (001) surface of the Fe_3_O_4_ crystal. The abundance of the (001) surface on Fe_3_O_4_ nanoparticles has been confirmed experimentally [74]. The Fe_3_O_4_ nanoparticle surface was modelled as a slab of $\left(\sqrt{2}\times \sqrt{2}\right)R45^{\circ }$ supercell of bulk cubic magnetite. The exact structure of (001) surface of magnetite is a delicate topic [75,76], sensitive to the chemical potential of species involved. We selected a stochiometric surface terminated with tetrahedrally coordinated Fe atoms (Fe_tet_), which is in line with previous computational studies [77,78,79] that identified this (001) surface and its termination as the most energetically favourable.

**Figure 7:**
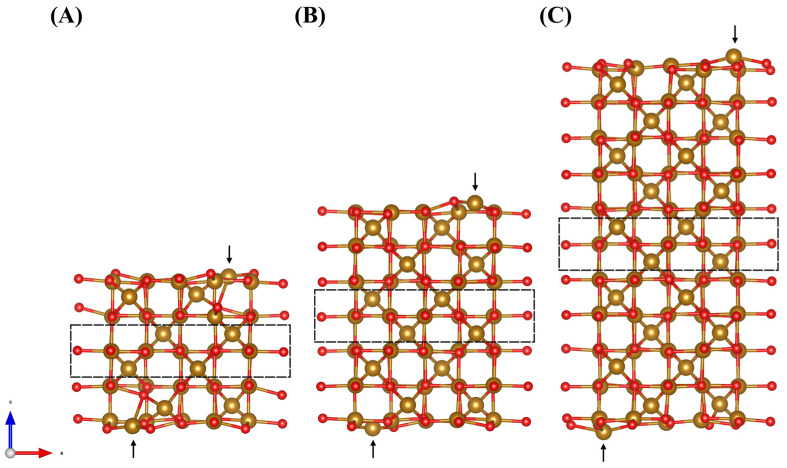
The 9-layer (**A**), 15-layer (**B**), and 23-layer (**C**) relaxed surfaces with Hubbard U correction. Dashed rectangle indicates region where atoms within are constrained to their relaxed bulk positions. The arrows point at Fe_tet_ atoms in the first surface termination layer.

Slabs of varying thicknesses (9, 15, and 23 layers) separated from its periodic image by a 25 Å vacuum layer were constructed to test for convergence of the surface energy (Figure 7). The resulting slabs were constrained by fixing the positions of the middle three layers of atoms at their relaxed bulk positions (the region enclosed by dashed lines in Figure 7) to simulate the bulk phase of Fe_3_O_4_, while the remaining outer layers of atoms were allowed to relax during optimization to simulate the surface reconstruction. The surface energy was calculated as

(1) $$\gamma =\frac{E_{slab}-NE_{bulk}}{2A}$$

where *E_slab_* is the total energy of the constructed surface slab, N is the equivalent number of Fe_3_O_4_ formula units in the slab, *E_bulk_* is the total energy per formula unit of the bulk Fe_3_O_4_, and A is the area of the surface slab. The calculated surface energies were compared to the surface energy found in a prior DFT study of Fe_3_O_4_ (001) surface [79]. These results can be seen in Table 2, where surface energy and Fe-O bond length are compared.

The bond length converges quickly as a function of the number of layers, while the surface energy converges very slowly. Due to computational constraints, we selected the 9-layer surface to investigate the physicochemical nature of the catechol adhesion. Figure 7 shows the final 9-layer force relaxed surface of which DHBA and BA adsorption will be modelled using DFT. One can see that the tetrahedrally coordinated Fe atoms on the top and bottom of the surface (shown by arrows) moved towards the bulk during relaxation, which is in agreement with literature [77].

### 2.3. DFT Analysis of DHBA and BA Adsorption at the (001) Surface

Previous adsorption studies [79,80,81] of single adatoms and water molecules on the surface of Fe_3_O_4_ have found favourable adsorption sites and have reported adsorption energies for the various species. Ni adatoms were found to adsorb favourably onto the (001) surface and reported an adsorption enthalpy of −3.21 eV [79]. This study also reported the incorporation of Ni and Ti into the subsurface by replacing Fe tetrahedral and octahedral sites, respectively. Incorporation energies of −3.39 eV for the Ni and −8.29 eV for the Ti were reported [79]. Gargallo-Caballero et al. [76] reported co-adatom adsorption as well as incorporation at octahedral/tetrahedral sites with energies of the order of –5.5 eV for the magnetite (001) surface. Additionally, adsorption enthalpies of −0.76 eV for H_2_O molecules adsorbing to surface Fe_tet_ atoms and −0.85 eV for single H atoms adsorbing to surface O atoms were reported [80,81]. For adsorption of DHBA onto the Fe_3_O_4_ surface, bonding to both tetrahedrally and octahedrally coordinated Fe atoms were considered, in a manner that maintained coordination seen in the bulk crystal.

**Figure 8:**
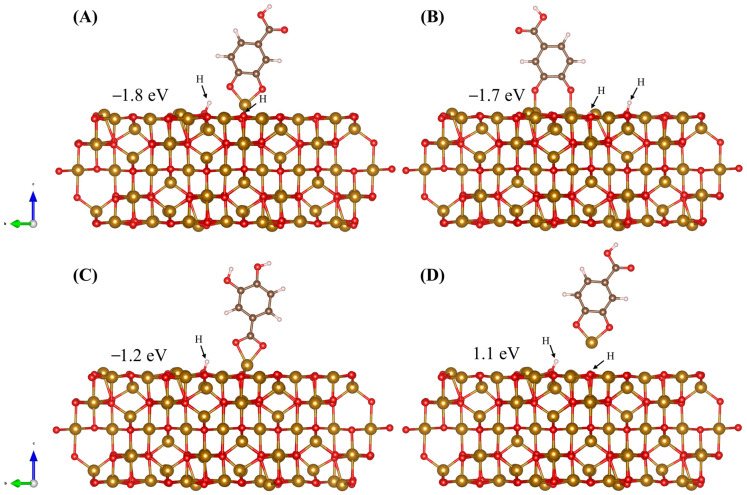
Adsorption of DHBA on the (001) surface of Fe_3_O_4_ in three different configurations, (**A**) chelating bidentate via phenolic OH groups, (**B**) bridging bidentate via phenolic OH groups, (**C**) chelating bidentate via carboxyl group, and (**D**) scenario where molecule adsorbs and detaches with Fe atom. The adsorption is accompanied by the surface adsorption of H^+^ ions displaced from OH groups. Values on each panel represent respective adsorption energies.

**Figure 9:**
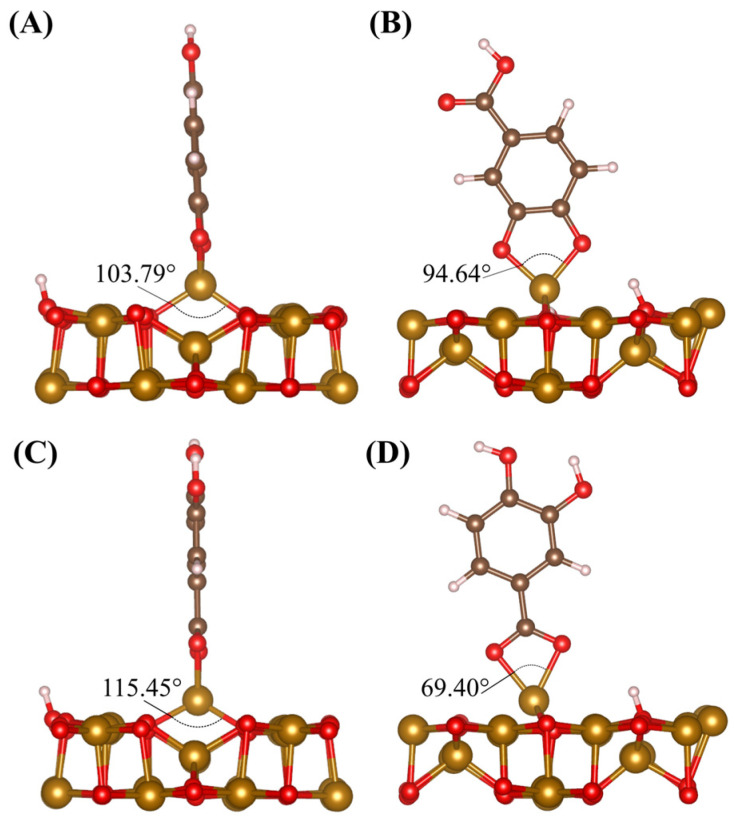
Surface Fe bond angles for (**A**) bottom of DHBA via phenolic OH group adsorption, (**B**) top of DHBA via phenolic OH group adsorption, (**C**) bottom of DHBA via carboxyl group adsorption, (**D**) top of DHBA via carboxyl group adsorption. Values of bond angles in degrees are shown on each panel.

Adsorption of DHBA is modelled on the (001) surface in four different configurations, seen in Figure 8. Two H atoms are cleaved from phenolic OH groups, in the case of Figure 8A,B,D, and are accommodated on surface O atoms. In the cases where the carboxyl group is responsible for adsorption (Figure 8C and Figure 9A), only one H atom is cleaved and accommodated by Fe_3_O_4_ forming a surface OH group. Adsorption strength of molecules to the surface of Fe_3_O_4_ is evaluated by the calculation of an adsorption enthalpy (*H_ads_*), which represents the difference between the total energy (*E_tot_*) of the adsorbed and nonadsorbed states

(2) $$H_{ads}=E_{tot}^{adsorbed}-E_{tot}^{nonadsorbed}$$

The nonadsorbed state is modelled as the surface slab with the respective molecule in a fixed, nonadsorbed state 10 Å above the surface in the vacuum layer. The calculated adsorption enthalpies are shown within Figure 8. The DFT study reveals that the three adsorbed configurations are energetically favourable, while the Fe_tet_-detached configuration (Figure 8D) is not. Figure 8A,C,D include adsorption of DHBA to a surface Fe_tet_ atom where Figure 8B captures adsorption of DHBA to two surface octahedrally coordinated Fe atoms (Fe_oct_). The chelating bidentate configuration is the most energetically favourable, closely followed by the bridging bidentate; the carboxyl bidentate configuration is the least favourable. These trends can be explained by examining the resulting bond angles. In the case of the chelating bidentate adsorption of DHBA via phenolic OH groups (Figure 8A), the Fe_tet_ atom is pulled away from the surface. This causes the bond angle between the surface Fe_tet_ atom and the surface O atom to change from 157.26° to 103.79°, seen in Figure 9A, returning the bond angle to a value much closer to what occurs in the bulk crystal (109.47°). This configuration also forms a bond angle with O atoms in the DHBA molecule of 94.64° (Figure 9B).

In the case where DHBA is adsorbed via carboxyl group, we again see the bond angles changing, this time to 115.45° between the surface O and Fe_tet_ and 69.40° between the O atoms in the carboxyl group and Fe_tet_, shown in Figure 9C,D, respectively. This less drastic change in the bond angle towards what is seen in the bulk crystal may contribute to the less favourable adsorption energy seen when adsorption occurs via the carboxyl group. Another consideration is that less energy may be required during the cleaving/adsorption process of the H atom in one of the phenolic OH groups (Figure 8A) compared to breaking the double bonded O in the carboxyl group (Figure 8C) to bond to the Fe_tet_ atom. We see similar changes in bond angle for adsorption via carboxyl group for BA to that of adsorption via carboxyl group for DHBA, this time changing to 114.33° in the case of Figure 9C and to 65.12° in the case of Figure 9D. This further explains the results seen experimentally, describing the role of phenolic OH groups for adsorption onto the Fe_3_O_4_ surface. In all configurations, the DHBA adsorption restores the coordination of the metal ions involved in bonding.

**Figure 10:**
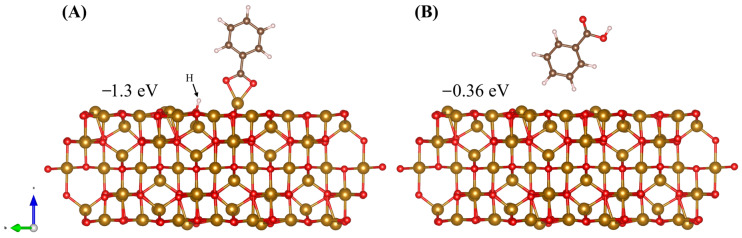
Adsorption of BA in two different configurations, (**A**) chelating bidentate via carboxyl groups, and (**B**) bonding of H to Fe_3_O_4_ surface O atoms. The adsorption can be accompanied by the surface adsorption of H^+^ ions displaced from OH groups.

Two different configurations of BA on the surface of Fe_3_O_4_ can be seen in Figure 10. In the chelating bidentate configuration (Figure 10A) adsorption energy is similar to that of the chelating bidentate adsorption via carboxyl group seen with DHBA (Figure 8C). In both cases where the carboxyl group is responsible for adsorption, the adsorption energy is much less than that of the chelating bidentate adsorption via phenolic OH groups. Figure 10B shows the optimized position of BA when it is positioned above the surface so that the H atoms point toward the surface. We can see that in this case adsorption is very weak. To explain the clear difference seen in the experimental results, where dispersion and capacitive properties are greatly improved with the use of DHBA, and the use of BA compares similarly to no dispersing agent at all; it is the electrostatic repulsion that must differ and not adsorption. For DHBA, adsorption utilizing the two adjacent OH groups results in the most energetically favourable adsorption configuration. Then, the carboxyl group is available to facilitate the electrostatic repulsion needed for good dispersion. However, in the case of BA, where the carboxyl functional group is involved in adsorption to the surface, there are no functional groups to provide the charge essential for electrostatic repulsion.

During the modelling of adsorption of both DHBA and BA, the presence of a solvent was neglected. Since the synthesis of Fe_3_O_4_ was conducted in a water medium, we would expect adsorption energies to reduce by approximately a factor of two [82] without changing trends. Thus, the presence of a solvent in our calculations would not affect the main conclusions, since we are interested in the trend of adsorption energy across different configurations and molecules, and not in the magnitude of adsorption energy seen.

## 3. Materials and Methods

### 3.1. Materials and Experimental Methods

Iron (II) chloride tetrahydrate, iron (III) chloride hexahydrate, ammonium hydroxide, 3,4-dihydroxybenzoic acid, benzoic acid, poly (vinyl butyral-co-vinyl alcohol-co-vinyl acetate) (PVB, MilliporeSigma, Canada), multiwalled carbon nanotubes (MWCNT, ID 4 nm, OD 13 nm, length 1–2 μm, Bayer, Leverkusen, Germany), and nickel foam (porosity 95%, thickness 1.6 mm, Vale, Toronto, ON, Canada), were used as starting materials.

Synthesis of Fe_3_O_4_ was performed by a chemical precipitation method [41,53] using aqueous solutions of iron (II) chloride and iron (III) chloride. The molar ratio of FeCl_2_ to FeCl_3_ in the solutions was 1:2. A similar procedure was carried with DHBA to analyse the influence of DHBA on the electrode performance, as well as with BA to gain insight into the dispersion and adsorption mechanisms involved. For the synthesis of Fe_3_O_4_ in the presence of MWCNT, a 3 g L^−1^ MWCNT suspension, containing 1.5 g L^−1^ DHBA or BA, was initially prepared. Preparation included ultrasonication of the MWCNT suspensions using a high energy Cole-Parmer (VCX 500, Cole-Parmer, Quebec City, QC, Canada) ultrasonic processor. Iron (II) chloride tetrahydrate and iron (III) chloride hexahydrate were added to the suspension, allowing for an Fe_3_O_4_:MWCNT mass ratio of 4:1. The pH of the solutions was adjusted to pH = 9 by ammonium hydroxide. Chemical precipitation was performed at 50 °C at continuous stirring. Obtained suspensions were filtrated, washed to remove non-adsorbed dispersant and reaction by-products, and dried overnight in an oven at 60 °C.

Electrodes were prepared by impregnation of Ni foam current collectors with slurries, containing Fe_3_O_4_, MWCNT and PVB binder. The mass ratio of Fe_3_O_4_:MWCNT:PVB was 80:20:3. The mass of the impregnated material after drying was 40 mg cm^−2^. The impregnated Ni foams were pressed to 30% of their original thickness in order to improve electrical contact of the impregnated material and current collector.

A Bruker Vertex 70 spectrometer (Bruker, Milton, ON, Canada) was used for the Fourier Transform Infrared Spectroscopy (FTIR) experiments. Zeta potential measurements were performed using a dynamic light scattering instrument (DelsaMax Pro: Beckman Coulter, Brea, CA, USA). Electrochemical studies were performed in aqueous 0.5 M Na_2_SO_4_ electrolyte using PARSTAT 2273 potentiostat (AMETEK, Berwyn, PA, USA) for cyclic voltammetry (CV) and electrochemical impedance spectroscopy (EIS). Galvanostatic charge–discharge (GCD) investigations were performed using Biologic VMP 300 potentiostat (BioLogic, Seyssinet-Pariset, France). Testing was performed using a 3-electrode electrochemical cell containing a working (impregnated Ni foam), counter-electrode (Pt mesh, MiliporeSigma, Oakville, ON, Canada), and a reference electrode(SCE, saturated calomel electrode, AMETEK, Berwyn, PA, USA). Mass and area normalized capacitances were calculated from the corresponding CV and GCD data, as described by previous studies [83,84]. The capacitances calculated from the CV and GCD data represented integral capacitances measured in a potential window of −0.8–0 V versus SCE. The capacitances calculated from the EIS data represented differential capacitances measured at an open circuit potential at voltage amplitude of 5 mV. CV results were obtained at 2, 5, 10, 20, 50, and 100 mV s^−1^ scan rates with EIS measurements performed afterwards. GCD results were obtained at 3, 5, 7, 10, 20, 30, and 40 mA cm^−2^ current densities.

### 3.2. Computational

The first-principles electronic structure calculations were performed in the framework of DFT [57] using Perdew–Burke–Ernzerhof (PBE) generalized gradient approximation [85] for the exchange correlation functional, augmented by the DFT-D3 correction with Becke–Johnson damping [86,87] to capture van der Waals interactions. The Vienna ab initio simulation program (VASP) (version 5.4.4, University of Vienna, Vienna, Austria) [88,89,90] and projector augmented-wave potentials [91] were used, where the *p* semi-core states were treated as valence states for Fe potentials in all calculations. Standard potentials were used for all other elements. The cut-off energy for a plane wave expansion of 500 eV was used for the relaxation of the bulk and surface structures to achieve an accurate stress tensor, and the cut-off energy of 400 eV was used in the case of adsorption calculations. During bulk relaxation, atomic position, cell shape, and cell volume were relaxed, whereas only atomic positions were relaxed during the surface and adsorption calculations. We included on-site Coulomb interaction to treat the highly correlated Fe 3*d*-electrons in the framework of Dudarev et al. [92] using an effective Hubbard energy of U = 3.7 eV [79]. Collinear spin-polarized calculations were performed for all structures. Magnetic moments were initialized with opposing spin orientations of magnitude 4.0 µ_B_ for tetrahedrally and octahedrally coordinated Fe atoms [93]. Full self-consistent structural optimization was performed for the bulk, while only forces were relaxed for surface models of Fe_3_O_4_ with additional constrains (see Section 2.2 for more details). The structure was considered as optimized when the magnitude of Hellmann–Feynman forces acting on atoms dropped below 50 meV/Å and components of the stress tensor did not exceed 1 kbar. The ground-state energy was calculated using first order Methfessel–Paxton smearing with a width of 0.02 eV. A blocked-Davidson algorithm with high precision is used during the relaxation of the bulk and surface Fe_3_O_4_ structures. The Brillouin zone was sampled with a Γ-centered k-mesh generated automatically with a linear density of 15 divisions per 1 Å^−1^ of the reciprocal space. Adsorption calculations utilize a preconditioned conjugate gradient with normal precision and automatic Γ-centered k-mesh with 30 divisions per 1 Å^−1^. Construction of the Fe_3_O_4_ (001) surfaces is discussed in the results and discussions section. All structure files and VASP input files used in this work can be found in the Supporting Information section. Structure files can be visualized in VESTA [94].

## 4. Conclusions

We have investigated the role DHBA has on the electrochemical performance of composite Fe_3_O_4_-MWCNT anode material for use in supercapacitors. DHBA’s exceptional adsorption and dispersing properties, utilized during synthesis of Fe_3_O_4_, contribute to the significant increase in capacitance at low values of impedance. With a mass loading of 40 mg cm^−2^, a capacitance of 4.59 F cm^−2^ was obtained from CV data at a scan rate of 2 mV s^−1^ and 4.72 F cm^−2^ from GCD data at a current density of 3 mA cm^−2^. DFT surface and adsorption study of DHBA and BA on the Fe_3_O_4_ (001) surface sheds light on the adsorption and dispersion mechanisms seen experimentally. The largest magnitudes of adsorption enthalpies, −1.8 and −1.7 eV, are seen for DHBA chelating bidentate and DHBA bridging bidentate configurations, respectively. Lower adsorption enthalpies were calculated for adsorption of DHBA and BA via bidentate bonding of the carboxyl group, where we see comparable adsorption enthalpies of −1.2 and −1.3 eV, respectively. One can therefore conclude that, due to favourable and comparable adsorption enthalpies of DHBA and BA utilizing a carboxyl functional group, the difference in dispersion and electrochemical performance seen when comparing these two molecules as dispersing agents is dependent on the means of electrostatic repulsion. We see that the more favourable adsorption configurations for DHBA are those that rely on the phenolic OH groups for adsorption, allowing the carboxyl group to facilitate electrostatic repulsion. The Fe_3_O_4_-MWCNT anode obtained with the use of DHBA as a dispersing agent, showing improved electrochemical properties, is favourable in the development of advanced energy storage devices.

## Figures and Tables

**Table 1:** Comparison of lattice parameter, O-Fe bond lengths, magnetic moments to previous DFT study of bulk Fe_3_O_4_ and experimentally reported values.

Parameter	Literature PBE+U [71]	This Work PBE+U+D3	Experimental
Lattice parameter (Å)	8.488	8.453	8.396 [72]
Bond length (oct) (Å)	2.09	2.07	2.07 [73]
Bond length (tet) (Å)	1.90	1.91	1.88 [73]
Magnetic moment (Fe_oct_) (μ_B_)	3.96	3.92	-
Magnetic moment (Fe_tet_) (μ_B_)	4.09	4.02	-
Magnetic moment (O) (μ_B_)	0.030	0.045	-
Total magnetic moment (μ_B_ /f.u.)	4.0	4.0	4.1 [72]

**Table 2:** Fe_3_O_4_ (001) surface energy and Fe-O bond length of surface tetrahedrally coordinated Fe, for 23-layer, 15-layer, and 9-layer slabs calculated at PBE+U+D3 level of theory and compared to previous surface study using PBE+U.

Surfaces (PBE+U+D3)	Surface Energy (J m^−2^)	Surface Fe-O Bond Length (Å)
23 layers	0.48	1.92
15 layers	0.73	1.81
9 layers	0.90	1.90
Literature, 9 layers (PBE+U)	0.96 [77]	1.89 [78]

## Data Availability

The data is available in this manuscript and Supplementary information.

## Supplementary Materials

The following supporting information can be downloaded at: https://www.mdpi.com/article/10.3390/molecules28041562/s1. This paper is accompanied by a ZIP archive file that contains VASP files (POSCAR) for all structures calculated in the present work. In addition, structure-specific input files (INCAR) as well as KPOINTS files are provided. POSCAR files can be visualized using VESTA [94].

## Sample Availability

Samples of Fe_3_O_4_ are available from the authors.
